# Recurrently Infected Rheumatoid Nodule Causing Posterior Interosseous Nerve Palsy: A Report of a Rare Case

**DOI:** 10.7759/cureus.87470

**Published:** 2025-07-07

**Authors:** Kazuhiro Ikeda, Hiromitsu Tsuge, Takamasa Kudo, Sho Kohyama, Takaji Yanai

**Affiliations:** 1 Department of Orthopedic Surgery, Institute of Medicine, University of Tsukuba, Tsukuba, JPN; 2 Department of Orthopedic Surgery, Kikkoman General Hospital, Noda, JPN

**Keywords:** infection, posterior interosseous nerve palsy, recurrent, rheumatoid nodule, surgery

## Abstract

Rheumatoid nodules are typically benign and asymptomatic, requiring no specific treatment. However, we encountered a diagnostically challenging case involving a painful elbow mass that rapidly enlarged. The lesion eventually caused posterior interosseous nerve (PIN) palsy. Surgical excision revealed histological features of a rheumatoid nodule with marked neutrophilic infiltration, raising suspicion of superimposed infection. Despite initial negative cultures, the lesion recurred aggressively, and infection was later confirmed by pathogen isolation. After adjusting immunosuppressive therapy and administering antibiotics, the patient achieved long-term remission with functional recovery. This case underscores the importance of distinguishing infection from rheumatoid activity in atypical nodular presentations.

## Introduction

Rheumatoid arthritis (RA) is a chronic inflammatory disease primarily affecting the synovium, and approximately 20-30% of patients develop rheumatoid nodules [1,2]. These nodules are typically asymptomatic and non-infectious. Therefore, conservative treatment is generally preferred, with the primary aim of controlling the underlying disease activity [3]. In cases where complications such as nerve compression, infection, or ulceration are present, surgical excision may be considered [3-7]. We encountered a rare case of an infected rheumatoid nodule complicating posterior interosseous nerve (PIN) palsy. The mass recurred shortly after the initial surgery, and the management was challenging due to concomitant infection. We report this unusual case, which offers important insights into treatment strategies.

## Case presentation

An 81-year-old woman with a history of giant cell arteritis and polymyalgia rheumatica had been receiving oral prednisolone at 7.5 mg/day. During routine outpatient follow-up with her rheumatologist, she developed a painful mass in the anterior aspect of the left elbow, along with elevated inflammatory markers (WBC 13,300/μL (normal range: 3,500-9,000/μL); CRP 8.2 mg/dL (normal range: <0.3 mg/dL)), and was hospitalized for further evaluation and treatment. Serum uric acid was not elevated (2.9 mg/dL (normal range: 4.0-7.0 mg/dL)). Her prednisolone dosage was increased to 20 mg/day, and azathioprine and tacrolimus were added. This resulted in a decrease in inflammatory markers (WBC 8,400/μL; CRP 3.5 mg/dL), and she was discharged. However, four months later, she developed left finger drop and was referred to our orthopedic department.

**Figure 1:**
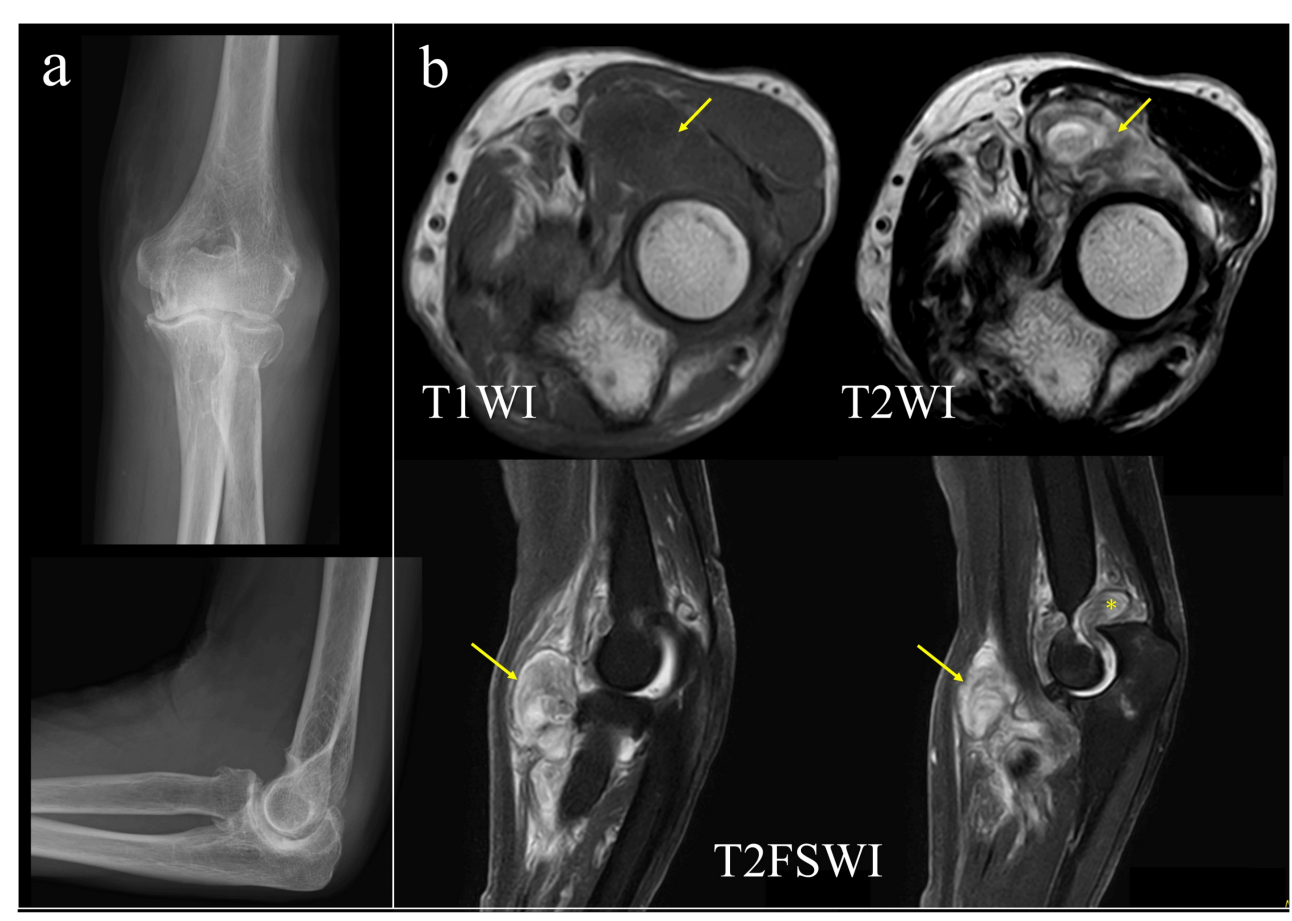
Initial imaging findings (a) Anteroposterior and lateral radiographs of the left elbow show mild joint space narrowing and bone erosion consistent with Larsen Grade 2. (b) MRI of the left elbow. Yellow arrows indicate the mass lesion, and (*) indicates enhanced synovial thickening in the elbow joint. MRI: Magnetic resonance imaging; T1WI: T1-weighted images; T2WI: T2-weighted images; T2FSWI: Fat-suppressed T2-weighted images

On initial examination, her left elbow was swollen, and a 4 cm tender mass was noted on palpation at the distal volar aspect of the elbow. Synovial fluid cultures were negative. Manual muscle testing (MMT) revealed Grade 2 weakness in muscles innervated by the PIN, including the extensor pollicis longus (EPL) and extensor digitorum communis (EDC), without any sensory deficits. Magnetic resonance imaging (MRI) revealed a mass extending from the humeroradial joint into the brachioradialis muscle, characterized by low signal intensity on T1-weighted images (T1WI) and high signal intensity on T2 fat-suppressed (T2FS) sequences, with surrounding inflammatory changes (Figure 1).

**Figure 2:**
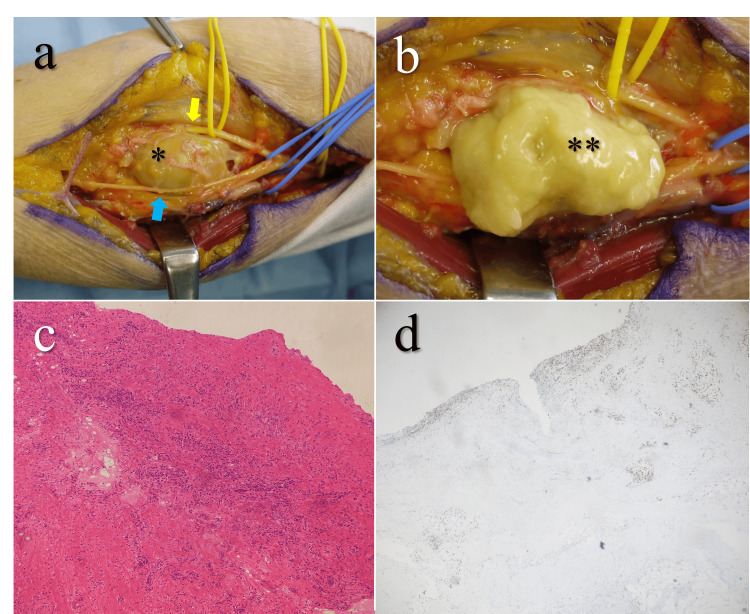
Intraoperative findings and histopathological examination at the first surgery (a) Intraoperative view. A well-encapsulated mass (*) was compressing the posterior interosseous nerve (yellow arrow) and the superficial branch of the radial nerve (blue arrow). (b) Intraoperative view. The interior of the mass capsule was filled with yellow necrotic tissue (**). (c) Histopathological image (hematoxylin and eosin staining). Neutrophilic infiltration and aggregation of macrophages are observed surrounding fibrinoid necrosis, raising suspicion of superimposed infection (×4 objective). (d) CD68 immunostaining shows palisading granulomas formed by macrophages, typical of a rheumatoid nodule (×4 objective).

One week after our initial evaluation, we performed surgical excision in response to the mass enlargement and worsening PIN palsy (Figure 2). Tacrolimus was discontinued prior to the first surgery due to concerns about postoperative infection, and prophylactic cefazolin was administered both before skin incision and for 24 hours postoperatively. We found an encapsulated mass filled with necrotic material compressing the posterior interosseous branch of the radial nerve. We resected the mass along with the synovium of the elbow joint and submitted specimens for histopathological and microbiological examination. Histopathological examination revealed central fibrinoid necrosis surrounded by CD68-positive macrophages arranged in a palisading pattern, consistent with a rheumatoid nodule. Accordingly, the patient was diagnosed with elderly-onset RA, despite negative results for both rheumatoid factor and anti-CCP antibodies. Additionally, the rheumatoid nodule exhibited marked neutrophilic infiltration within the area of fibrinoid necrosis. Despite negative results from microbiological stains (periodic acid-Schiff, Gram, Grocott) and interferon-γ testing, superimposed infection could not be excluded.

**Figure 3:**
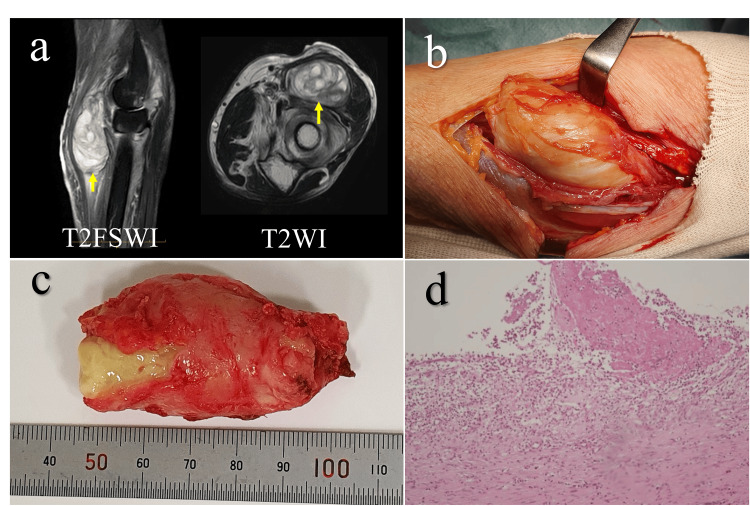
Intraoperative findings at revision surgery for recurrent mass (a) MRI performed after the initial surgery shows a recurrent mass within the brachioradialis muscle (yellow arrows). (b) Intraoperative view. A yellowish-white mass located within the brachioradialis muscle was excised. (c) Resected specimen. Similar to the initial surgery, the mass is encapsulated and filled with yellowish-white necrotic material. (d) Histopathological image (hematoxylin and eosin staining). The lesion contains fibrin and exudate. A small number of histiocytes are present within the cyst wall, which is composed of inflammatory granulation tissue. However, the palisading arrangement is immature and poorly defined (×10 objective). MRI: Magnetic resonance imaging; T2FSWI: Fat-suppressed T2-weighted images; T2WI: T2-weighted images

At one month postoperatively, the tender mass recurred at the same site and enlarged to 5 cm in diameter, and improvement in PIN palsy remained limited. Seven weeks after the initial operation, a second surgery was performed to remove the recurrent mass (Figure 3). We considered the possibility that prophylactic antibiotic administration had contributed to the negative culture results at the initial surgery and therefore administered cefazolin after mass excision during the second surgery. The histological examination revealed immature palisading granulomas, suggesting that the lesion rapidly developed under acute inflammatory conditions. Methicillin-sensitive *Staphylococcus aureus *and *Escherichia coli* were isolated from the resected mass, confirming the presence of infection.

**Figure 4:**
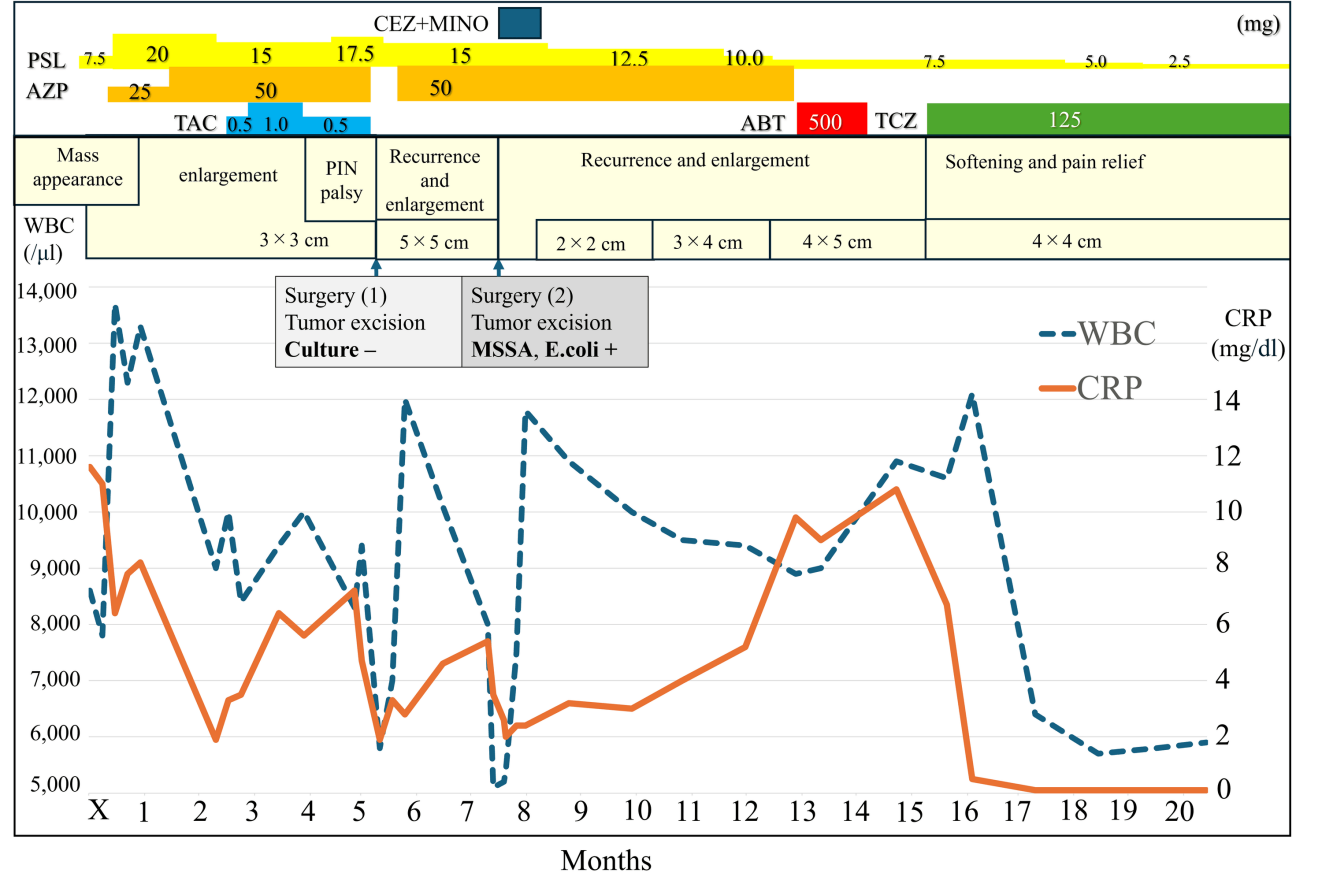
Clinical summary of the present case The top panel shows the medication timeline, the middle panel depicts chronological changes in the mass lesion, and the bottom panel shows changes in inflammatory markers over time. X indicates the onset of posterior interosseous nerve palsy. CEZ: Cefazolin; MINO: Minocycline; PSL: Prednisolone; AZP: Azathioprine; TAC: Tacrolimus; ABT: Abatacept; TCZ: Tocilizumab; MSSA: Methicillin-sensitive *Staphylococcus aureus*; E.coli: *Escherichia coli*

Prednisolone was tapered, and a one-month course of cefazolin and minocycline was administered (Figure 4). However, over the six months following the second surgery, inflammatory markers gradually worsened (WBC 8,900/μL; CRP 9.8 mg/dL), and the mass showed slow re-enlargement to 4 × 5 cm. Although infection could not be completely ruled out, the relatively indolent clinical course suggested inadequate control of RA, prompting the addition of immunosuppressive therapy. Abatacept was initiated; however, it proved ineffective, and tocilizumab was subsequently introduced eight months after the second surgery. Following tocilizumab initiation, the inflammatory response normalized rapidly, the mass softened, and tenderness improved. Clinical remission was maintained for over two years without recurrence. The PIN palsy also gradually improved, and final strength in the EPL and EDC reached MMT Grade 4.

## Discussion

The key clinical question in this case was whether the mass enlargement was attributable to infection or to insufficient control of RA. This distinction was essential because the therapeutic approaches differ fundamentally. Infection requires a reduction in immunosuppressive therapy, whereas uncontrolled RA calls for its intensification. To aid this differentiation, we focused on two clinical indicators: the presence of localized tenderness and the rate of mass enlargement.

Rheumatoid nodules are generally asymptomatic and non-infectious. Histologically, they resemble caseating granulomas of tuberculosis, consisting of aggregated macrophages and lymphocytes driven by chronic inflammation [1-3,8-10]. Because they typically lack neutrophilic infiltration, the hallmark of acute inflammation, they are usually non-tender [1-3]. In contrast, the present case exhibited localized tenderness at initial presentation. The presence of tenderness in this case may reflect acute inflammation, as early-stage rheumatoid nodules sometimes exhibit prominent neutrophilic infiltration and are associated with pain [11]. Therefore, tenderness on examination could serve as a clinical indicator of acute inflammation, including possible superimposed infection.

To further evaluate whether this acute inflammatory reaction was due to infection, we considered the rate of mass enlargement. Postoperative recurrence of rheumatoid nodules is reported to be associated with systemic disease activity [3,10,11] and occurs in more than half of cases within five years postoperatively [12-15]. However, recurrence accompanied by rapid growth, as observed in the present case, is extremely rare. The histological findings at the time of the second surgery showed immature palisading granulomas, suggesting that the lesion had formed rapidly in the context of acute inflammation. In contrast, the recurrence that occurred after antibiotic treatment was associated with only mild pain and progressed slowly. This presentation was more consistent with a local manifestation of uncontrolled RA. Indeed, after resuming immunosuppressive therapy, the mass softened and the pain subsided. These findings underscore the need to carefully evaluate the potential contribution of infection to local disease activity in RA. A rapidly enlarging tender mass should raise suspicion for underlying infection, often warranting surgical excision for definitive diagnosis. In such cases, prophylactic antibiotics should ideally be administered after mass removal to preserve the diagnostic yield of microbiological cultures.

Additionally, this case involved PIN palsy caused by the rapid expansion of the infected nodule. PIN palsy is a rare complication of RA, with only 72 cases reported in the literature [16,17]. Known etiologies include vasculitis associated with RA, drug-induced neuropathy, and mechanical compression by hypertrophied synovium or radial head subluxation at the arcade of Frohse [16,17]. Therefore, accurate identification of the underlying cause is essential when PIN palsy arises in RA patients. In the present case, PIN palsy was clearly caused by compression from the infected rheumatoid nodule. We performed prompt surgical decompression followed by pathological and microbiological analyses of the resected specimen. These investigations enabled a definitive diagnosis and guided appropriate medical management, which led to a favorable recovery of nerve function.

This case report has several limitations. First, some of the clinical indicators we used to differentiate infection from rheumatoid activity, such as localized tenderness and rate of mass enlargement, are inherently subjective and lack standardized diagnostic criteria. Second, although we attempted to exclude persistent infection based on clinical course and microbiological results, we cannot completely rule out the possibility of subclinical or low-grade infection, especially given the patient’s immunosuppressed state. Third, as this is a single case report, the generalizability of our observations is limited. Accumulation of similar cases is required to validate these clinical observations and to develop more objective diagnostic guidelines.

## Conclusions

This case highlights the importance of distinguishing superimposed infection from active RA in clinical practice. Rapid enlargement of a nodule accompanied by localized tenderness may indicate underlying infection. In such situations, prompt surgical intervention for tissue sampling can be critical not only for detecting infection but also for establishing the diagnosis of RA itself. Accumulation of similar cases may help establish more reliable clinical criteria for differentiating infection from active RA and optimizing therapeutic strategies.
